# Long-Term Characteristics of Severe COVID-19: Respiratory Function, Functional Capacity, and Quality of Life

**DOI:** 10.3390/ijerph19106304

**Published:** 2022-05-23

**Authors:** Ukbe Sirayder, Deniz Inal-Ince, Busra Kepenek-Varol, Cihangir Acik

**Affiliations:** 1 Department of Physiotherapy and Rehabilitation, Faculty of Health Sciences, Nuh Naci Yazgan University, Kayseri, Kocasinan 38170, Turkey; busrakepenek@gmail.com (B.K.-V.); acik@nny.edu.tr (C.A.); 2 Faculty of Physical Therapy and Rehabilitation, Hacettepe University, Ankara, Samanpazari 06100, Turkey; dince@hacettepe.edu.tr

**Keywords:** lung function, post-COVID-19, fibrosis, functional capacity, fatigue, quality of life

## Abstract

Recovery from pneumonia takes around 3–6 months in individuals with severe COVID-19. In order to detect the isolated damage caused by COVID-19, the 6-month period must pass after the recoveries. However, to our knowledge, no published study analyzes a comprehensive evaluation of individuals with severe COVID-19 after 6 months. We aimed to evaluate long-term consequences of severe COVID patients by comparing respiratory function, functional capacity, quality of life, fatigue, and balance 6 months after the intensive care unit (ICU) discharge with healthy individuals. Method: 26 post-COVID adult patients and 26 healthy individuals (control group) were included in this study. Physical characteristics of both groups and patients’ ICU data, including APACHE II scores, were recorded. Lung function, respiratory, and peripheral muscle strength were measured. The lower limit of normal (LLN) cutoff points for forced vital capacity (FVC) and forced expiratory volume in one second (FEV_1_) were calculated. A 6-minute walk test (6MWT) was used to assess functional capacity. Time Up and Go test (TUG) with a stadiometer was performed for balance evaluation. Quality of life was evaluated using Nottingham Health Profile (NHP) and St George Respiratory Questionnaire (SGRQ). Results: Percent predicted FVC and FEV_1_, 6MWT distance, change in oxygen saturation (SpO_2_) during 6MWT, were lower and NHP, SGRQ, FSS scores and TUG findings were higher in the COVID group than the control group (*p* < 0.05). The FVC of nine individuals and the FEV_1_ value of seven individuals in the COVID-19 group were below the LLN values. A moderate correlation was found between ICU length of stay and APACHE II scores with FVC, FEV_1_, 6MWT distance, and change in SpO_2_ values in the COVID-19 patients (*p* < 0.05). Conclusion: Respiratory function, functional capacity, quality of life, and fatigue levels of the individuals with severe COVID-19 infection are impaired at 6 months after ICU discharge. Impaired lung function might be associated with severe inflammation, which starts during the acute infection process and the fibrous tissue during the healing process, impairing lung compliance and diffusion capacity. Infiltration of coronavirus and inflammatory cytokines into the cerebrum and muscle might have increased fatigue and decreased functional capacity. Overall, our study suggests that severe COVID patients need post-discharge care even after 6 months of recovery.

## 1. Introduction

The new Coronavirus (SARS-CoV-2) causes gradually worsened respiratory problems, acute respiratory distress syndrome (ARDS), septic shock, and/or multi-organ failure, requiring prolonged mechanical ventilation support in 5% of these COVID-19 cases [1]. In addition to respiratory problems, cardiovascular, neurological, musculoskeletal and gastrointestinal system effects, especially in children [2], can also be accompanied in individuals with COVID-19 infection. In particular, patients with invasive mechanical ventilation (IMV) and vasopressor therapy for hypotension and acute hypoxemic respiratory failure are hospitalized and followed up in the intensive care unit (ICU). Circulatory parameters are affected, and vascular inflammation and coagulation disorders can be seen in individuals with advanced age and comorbidities [3]. Negative hematological changes including increased inflammatory cytokines such as tumor necrosis factor-alpha (TNF-alpha), interferon-gamma (IFN-γ), and interleukin 6 (IL-6) are prominent in cases with severe infection [4,5]. Systemic inflammation caused by the virus also disrupts the coagulant-anticoagulant balance [6] and increases the frequency of acute cerebrovascular diseases, impaired consciousness, and skeletal muscle damage in severe cases of COVID-19 disease [5].

There may be functional loss in patients discharged from ICU after COVID-19 pneumonia due to acute illness, ARDS, and intensive care processes in the post-acute period. Permanent pulmonary damage (alveolar endothelial dysfunction and accompanying pulmonary fibrosis), polyneuropathy, myopathy, weakness, loss of muscle mass, pain, fatigue, depression, anxiety, and occupational problems are frequently observed in hospitalized COVID-19 patients [7,8,9].

Development of a restrictive type of lung disease and reduced diffusion capacity often draws attention to lung function during the post-COVID period, with an average of 1 month after discharge from the hospital or symptoms resolved [10]. Inflammatory biomarker levels return to normal levels in the post-COVID 3-month period, but the damage in the lung continues for more extended periods. Computed tomography (CT) examinations performed 3 months later show an interstitial pattern, fibrosis, and consolidation [11]. It takes 5–6 months for pneumonia to be wholly absorbed, and pulmonary fibrosis develops [12]. The permanent sequelae are not associated with aerobic capacity or respiratory function; they need to be explained by different mechanisms [13].

In order to detect the isolated damage caused by COVID-19, the 6-month period must pass after the recovery. However, today, to our knowledge, there are no studies in the literature that provide a comprehensive assessment of the mechanisms of persistent sequelae in individuals with severe COVID-19 after 6 months. Therefore, our study aimed to evaluate respiratory function, functional capacity, peripheral muscle strength, balance, physical activity, and quality of life in patients with severe COVID-19 infection to give a comprehensive overview of the patients 6 months after severe COVID-19. We evaluated individuals at the 6th month after discharge to show the effect of pulmonary fibrosis alone, which is likely to develop after pneumonia in the lung tissue. In addition, we aimed to investigate whether the length of stay in the ICU and physiological characteristics, including Acute Physiology Chronic Health Evaluation (APACHE) II scores, are related to respiratory function, functional capacity, peripheral muscle strength, balance, physical activity, and quality of life in the COVID-19 group.

## 2. Materials and Methods

### 2.1. Participants

A total of 52 individuals, 26 with COVID-19 and 26 healthy subjects, were included in the study. We calculated (G*Power Version 3.1.9.4, Franz Faul, Universitat Kiel, Düsseldorf, Germany) a power of 80% with 0.05 significance, a difference to be detected of 55 m, and a standard deviation of 82 m in 6-minute walk test (6MWT) distance [14], generating a sample of 26 individuals per group.

All individuals’ sociodemographic information and vaccination status were recorded. ICU characteristics, including type of mechanical ventilation (non-invasive or invasive), corticosteroid therapy, blood laboratory results, and APACHE II scores, were recorded after patients were hospitalized. The data were collected from March 2021 to January 2022. COVID-19 pneumonia patients followed up for at least 24 h in the ICU and in the post-COVID 6-months, who did not have any other acute diseases (such as infection and trauma) that would prevent performing the tests and/or interfering with the test results, who could cooperate with the tests to be performed, and who volunteered to participate in the study were included to the COVID-19 group. Individuals with pulmonary, orthopedic, neurological, vestibular, and psychological problems diagnosed before COVID-19 infection; myocarditis developed after COVID-19 infection; and those pregnant were excluded. Age and gender-matched healthy individuals without any acute diseases (such as infection, trauma) that would prevent them from performing the tests and/or that could affect the test results and who were cooperative in the tests to be performed were included in the control group. Individuals who had chronic orthopedic, pulmonary, neurological, and psychological problems preventing them from performing the tests and those pregnant were excluded.

### 2.2. Experimental Protocol

First, sociodemographic information of all individuals included in the study was obtained, and all questionnaires were conducted. After the questionnaires were performed, balance evaluation, pulmonary function test, respiratory muscle strength measurement, peripheral muscle strength measurement, Time-Up Go (TUG) test, and a 6MWT were performed.

#### 2.2.1. Modified Medical Research Council (MMRC) Dyspnea Scale

Shortness of breath was evaluated using the Modified Medical Research Council (MMRC) dyspnea scale. It is a categorical scale in which individuals choose the most appropriate of the five expressions of dyspnea, between 0–4 points, to define their dyspnea levels [15].

#### 2.2.2. Lung Function Test

Spirometry (Cosmed Pony FX Spirometer, Milan, Italy) was used to measure forced vital capacity (FVC), forced expiratory volume in one second (FEV_1_), FEV_1_/FVC, peak expiratory flow rate (PEF), and forced expiratory volume from 25–75% (FEF_25–75%),_ based on the European Respiratory Society/American Thoracic Society (ERS/ATS) criteria. The test was performed in the sitting position. At least three technically acceptable measurements were obtained between the two best-measured FEV_1_ values, with no more than a 5% difference, and the best FEV_1_ value was selected for analysis. The lower limit of normal (LLN) for FVC and FEV_1_ was calculated for each subject [16].

#### 2.2.3. Respiratory Muscle Strength

Respiratory muscle strength was measured (Cosmed Pony FX Spirometer, Milan, Italy). For the measurement of maximum inspiratory pressure (MIP), maximum expiration was performed to the person, and immediately the respiratory tract was closed with a valve, and then the person was asked to perform maximum inspiration for 1–3 s. For maximum expiratory pressure (MEP), a maximal inspiration was performed, and then the person was asked to perform a maximal expiration of 2 s against the closed airway. At least three technically acceptable maneuvers were performed, with no more than a 5% difference between the two best-measured values [17].

#### 2.2.4. Peripheral Muscle Strength

Peripheral muscle strength was determined by measuring handgrip strength and quadriceps muscle strength using a digital dynamometer (Jtech Commander Muscle Tester, Midvale, UT, USA). The mean values of the right and left side measurements were obtained. Then, the measurements were recorded in Newton (N) using each side’s measurements [14].

#### 2.2.5. Evaluation of Functional Capacity

The functional capacity of individuals was evaluated using the 6MWT [18]. Before and after 6MWT, heart rate, blood pressure, oxygen saturation (SpO_2_) (Cosmed Spiropalm 6MWT, Rome, Italy), respiratory frequency were assessed, and fatigue levels during exertion, and dyspnea were evaluated using the Modified Borg Scale [19].

#### 2.2.6. Balance Evaluation

The stabilometer device (HUR Smartbalance 2031, Kokkola, Finland) to be used in the study is a balance mechanism that can move forward-backward, right-left, and 3D in the transverse plane. The device’s reliability has been demonstrated in different studies [20,21]. In addition, the Time-Up Go (TUG) test was used to assess balance. The TUG test is predicted to be an easy-to-apply and reliable test evaluating balance [22].

#### 2.2.7. Hospital Anxiety and Depression Scale (HADS)

Hospital Anxiety and Depression Scale (HADS) is a frequently used scale that the patient fills in themself, in which anxiety and depression symptoms are screened. Our study used the validated and reliable form of HADS adapted to Turkish society [23].

#### 2.2.8. Mini-Mental State Test

Mini-Mental State Test (MMST) was used to evaluate the cognitive functions of the patients. The MMST is a widely used and validated test in evaluating cognitive status [24].

#### 2.2.9. Fatigue Severity Scale (FSS)

Fatigue level of individuals was evaluated using FSS. In our study, the Turkish version of the FSS was used [25].

#### 2.2.10. International Physical Activity Questionnaire (IPAQ) Short Form

IPAQ short form was used to assess participants’ physical activity levels. Severe, moderate-severe, and walking include seven questions that question the time elapsed while doing these, and sitting time is considered a separate question. Metabolic equivalence (MET-minute) score is obtained as a result of calculations. Total MET values are calculated, and those with a total MET value of <600 MET-min/week inactive, 600–3000 MET-min/week of minimum active, and >3000 MET-min/week are classified as very active [26].

#### 2.2.11. St. George Respiratory Questionnaire (SGRQ) and Nottingham Health Profile (NHP)

SGRQ and NHP for quality of life were used. The SGRQ is a disease-specific quality of life questionnaire consisting of 76 items: the symptoms part (29 items), the activity part (9 items), and the impact part (38 items) [27]. The NHP is a frequently used measurement to assess the perceived health status. People’s problems in daily life are questioned in seven sub-categories: pain, emotional reactions, sleep, social isolation, physical activity, and energy [28].

### 2.3. Statistical Analysis

Statistical analyses and graphs were performed using SPSS Statistics 22.0 (IBM Inc., Armonk, NY, USA) and GraphPad Prism 9.0.0 (GraphPad Software, San Diego, CA, USA). Values are presented as the mean and related standard deviation, median values with minimum and maximum, frequencies, and percentages. The Kolmogorov–Smirnov test was applied to determine the compatibility of the parametric data with the normal distribution. Student’s *t*-test and the Mann–Whitney U test were used to compare the measured values of COVID-19 and control groups, as appropriate. Spearman correlation analysis was performed to examine the relationship between variables of the COVID-19 group. The descriptive level of significance was set at *p* < 0.05.

## 3. Results

### 3.1. Participant Characteristics

Participants’ characteristics are summarized in Table 1. Age, height, weight, body mass index, and smoking exposure were similar between COVID-19 and control groups (*p* > 0.05). There were 10 active smokers in the COVID-19 group and 7 in the control group. The MMRC score of the COVID-19 group was found to be significantly higher than that of the control group (*p* < 0.05). When the individuals in the COVID-19 group were examined, six individuals had never been vaccinated, two individuals had received a single dose, 11 individuals had received two doses, and seven individuals had received three doses. Of these, 13 were Sinovac (CoronaVac, Inactivated, Sinovac Life Sciences, Beijing, China) and 9 were BioNTech (BNT162b2 Vaccine, BioNTech SE, Mainz, Germany) vaccines. All of the individuals in the COVID-19 group were given azithromycin as antibacterial, enoxaparin sodium and acetylsalicylic acid as anticoagulant, and favipiravir as antiviral. Prednisolone and prednisone were given to 15 patients as steroids. In addition, the most common comorbidities encountered were hypertension and anxiety. After discharge, it was determined that four patients were diagnosed with hypertension and six patients were diagnosed with anxiety.

### 3.2. Lung Function Test

**Figure 1:**
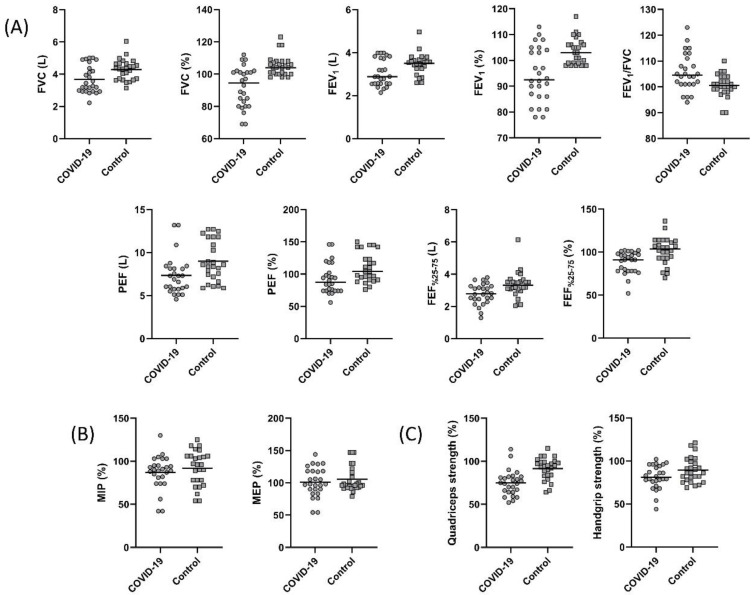
Comparison of (**A**) forced vital capacity (FVC) (*p* = 0.005), percent predicted FVC (*p* < 0.001), forced expiratory volume in one second (FEV_1_) (*p* = 0.04), percent predicted FEV_1_ (*p* = 0.001), FEV_1_/FVC (*p* = 0.255), peak expiratory flow (PEF) (*p* = 0.01), percent predicted PEF (*p* = 0.007), forced expiratory volume from 25 to 75% (FEF_25–75%_) (*p* = 0.01), percent predicted FEF_25–75%_ (*p* = 0.001), (**B**) percent predicted maximum inspiratory pressure (MIP) (*p* = 0.38), percent predicted maximum expiratory pressure (MEP) (*p* = 0.46), (**C**) percent predicted quadriceps strength (*p* < 0.001), and percent predicted handgrip strength (*p* = 0.048) between the COVID-19 and control groups.

The pulmonary function test parameters are summarized in Figure 1. A statistically significant decrease in FVC, FEV_1_, PEF, and FEF_25–75%_ was found in the COVID-19 group (*p* < 0.05, Figure 1A). The FEV_1_/FVC values were 104.50 (94.00–123.00) and 100.50 (90.00–110.00) in the COVID-19 and control groups, respectively. Although not statistically significant, the FEV_1_/FVC was low in the COVID-19 group (*p* > 0.05). The LLN for FVC was 3.51 ± 0.58 L and 3.52 ± 0.54 L for the COVID-19 and the control groups, respectively (*p* = 0.959). The LLN for measured FEV_1_ was 2.70 ± 0.44 L and 2.74 ± 0.42 L for the COVID-19 and the control groups, respectively (*p* = 0.729). The measured FVC value of nine individuals in the COVID-19 group and the measured FEV_1_ value of seven individuals were below the LLN values of measured FVC and FEV_1_. However, there was no individual in the control group with FVC and FEV_1_ values below the LLN value.

### 3.3. Respiratory and Peripheral Muscle Strength

Respiratory and peripheral muscle strength values are shown in Figure 1. MIP and MEP were similar between the groups (*p* > 0.05, Figure 1B). Handgrip strength and quadriceps muscle strength values were significantly lower in the COVID-19 group (*p* < 0.05, Figure 1C). The 15 (57.6%) subjects from the COVID-19 group had quadriceps strength lower than the 95% confidence interval of the controls.

### 3.4. Functional Capacity Evaluation

**Figure 2:**
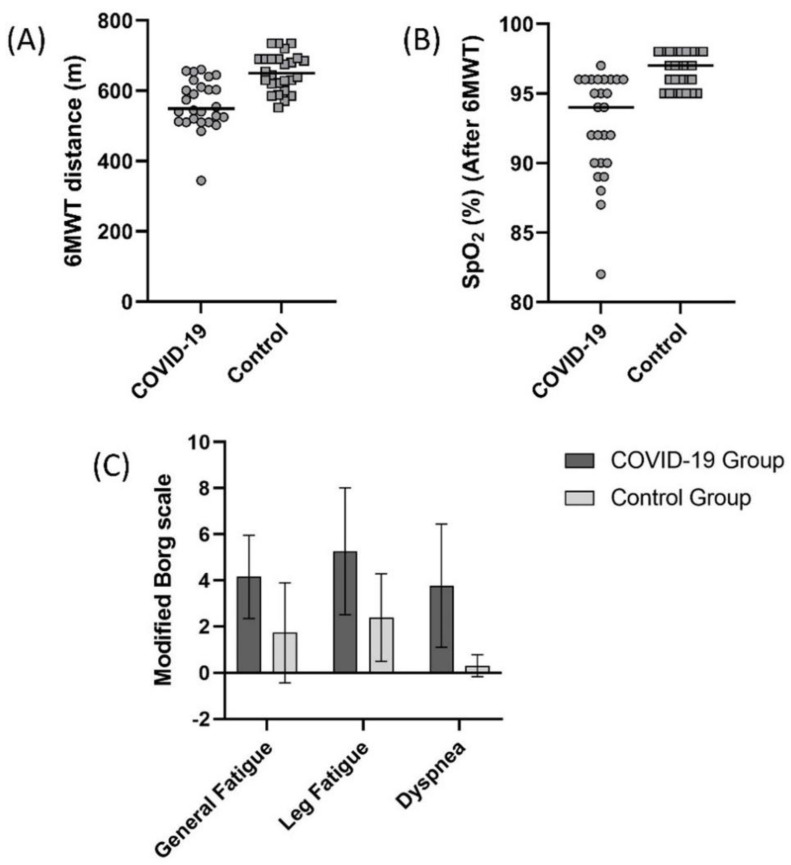
Comparison of (**A**) 6 minute walk test (6MWT) distance (*p* < 0.001), (**B**) oxygen saturation (SpO_2_) after 6MWT (*p* < 0.001), (**C**) general fatigue (Borg) (*p* < 0.001), leg fatigue (Borg) (*p* < 0.001), and dyspnea (Borg) (*p* < 0.001) scores between the COVID-19 and control groups.

A comparison of 6MWT distance values in the COVID-19 and control group is summarized in Figure 2. In the COVID-19 and control groups, 6MWT distance was 561.1 ± 71.0 m and 652.6 ± 53.4 m, respectively (Figure 2A, *p* < 0.05). The 6MWT distance of the 21 (80.7%) subjects from the COVID-19 group was lower than the controls’ 95% confidence interval (631.0–674.1 m). SpO_2_ value measured during 6MWT was significantly lower (Figure 2B), and general fatigue, leg fatigue, and dyspnea scores measured were significantly higher in the COVID-19 group after the test (Figure 2C, *p* < 0.05).

### 3.5. Balance Evaluation

**Figure 3:**
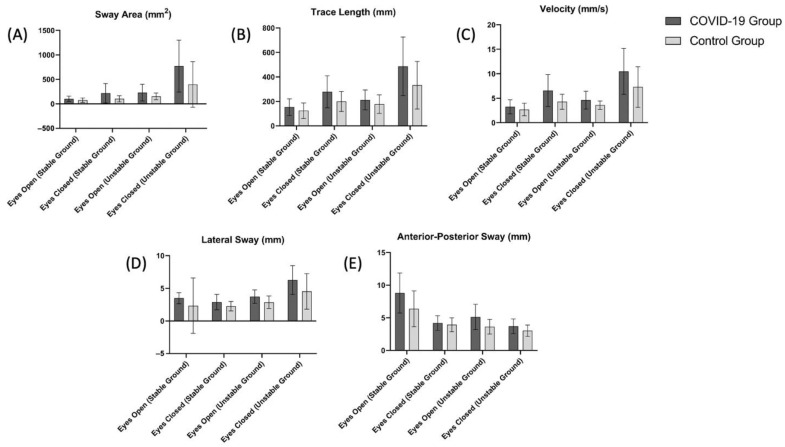
Comparison of (**A**) sway area (eyes open (stable ground), *p* = 0.049; eyes closed (stable ground), *p* = 0.009; eyes open (unstable ground), *p* = 0.038; eyes closed (unstable ground), *p* = 0.010), (**B**) trace length (eyes open (stable ground), *p* = 0.11; eyes closed (stable ground), *p* = 0.01; eyes open (unstable ground), *p* = 0.11; eyes closed (unstable ground), *p* = 0.01), (**C**) velocity (eyes open (stable ground), *p* = 0.14; eyes closed (stable ground), *p* = 0.003; eyes open (unstable ground), *p* = 0.01; eyes closed (unstable ground), *p* = 0.01), (**D**) lateral sway (eyes open (stable ground), *p* = 0.18; eyes closed (stable ground), *p* = 0.02; eyes open (unstable ground), *p* = 0.003; eyes closed (unstable ground), *p* = 0.01), (**E**) anterior-posterior sway eyes open (stable ground), *p* = 0.02; eyes closed (stable ground), *p* = 0.002; eyes open (unstable ground), *p* = 0.39; eyes closed (unstable ground), *p* = 0.004) scores between the COVID-19 and control groups.

The TUG test results and the balance evaluation using a stabiliometer are summarized in Table 2 and Figure 3, respectively. The TUG test scores of the COVID-19 group were significantly higher than the control group (*p* < 0.05, Table 2). Based on the scores obtained in the stabiliometer, the sway area (mm^2^) (Figure 3A) value measured for eyes open (stable ground and unstable ground) and eyes closed (stable ground, unstable ground) was significantly higher in the COVID-19 group (*p* < 0.05). Trace length (mm) (Figure 3B), velocity (mm/s) (Figure 3C), and lateral sway (mm) (Figure 3D) scores were significantly higher in the COVID-19 group in all measured conditions except for eyes open (stable ground) (*p* < 0.05).

### 3.6. Hospital Anxiety and Depression Scale (HADS), Fatigue Severity Scale (FSS), and Mini-Mental Test (MMST)

HADS, FSS, and MMST scores are shown in Table 2. HADS and FSS scores were found to be significantly higher in the COVID-19 group (*p* < 0.05), while there was no statistical difference between the groups in MMST scores (*p* > 0.05).

### 3.7. International Physical Activity Questionnaire (IPAQ) Short Form, St George Respiratory Questionnaire (SGRQ), and Nottingham Health Profile (NHP)

No significant differences were found in the IPAQ high and medium intensity scores, IPAQ walking and sitting score, and the IPAQ total score between the groups (*p* > 0.05, Table 2). The total score, symptom, activity, and impact scores of SGRQ were significantly higher in the COVID-19 group (*p* < 0.05). The total scores, pain, emotional reactions, sleeping, social isolation, physical activity, and energy scores of NHP were statistically significantly higher in the COVID-19 group (*p* < 0.05).

### 3.8. Conditions of Those Receiving Corticosteroid and Mechanical Ventilation in the COVID-19 Group

Comparison of respiratory function and functional capacity of the individuals with COVID-19 compared to those who received and did not receive steroid therapy and those who received non-invasive mechanical ventilation (NIMV) and the IMV treatment are summarized in Table 3. The percent predicted FVC and FEV_1_, FEV_1_/FVC, 6MWT distance (m), SpO_2_ (after 6MWT), length of stay in the ICU, and APACHE II values of those who received steroid treatment were found to be significantly lower than those who did not receive steroid treatment (*p* < 0.05). Similarly, the same parameters except for the length of stay in the ICU and APACHE II in individuals treated with the IMV were statistically significantly lower than those treated with NIMV (*p* < 0.05).

### 3.9. Relationship between the Length of ICU Stay, APACHE II, and TUG Scores with the Measured Parameters

Correlation of the length of ICU stay, APACHE II, and TUG scores of the individuals in the COVID-19 group with the measured parameters are shown in Table 4. A correlation was found between the length of stay in the ICU and vaccination status (r = −0.633, *p* < 0.001), percent predicted FVC (r = −0.564, *p* = 0.003), percent predicted FEV_1_ (r = −0.453, *p* = 0.02), 6MWT distance (r = −0.427, *p* = 0.03), the change in SpO_2_ (r = 0.469, *p* = 0.02), HADS (r = 0.394, *p* = 0.047), SGRQ symptom (r = 0.551, *p* = 0.004), SGRQ activity (r = 0.532, *p* = 0.005), SGRQ impact (r = 0.573, *p* = 0.002), and SGRQ total scores (r = 0.568, *p* = 0.002). However, no significant correlation was found between TUG (r = −0.095, *p* = 0.64), MMRC (r = 0.327, *p* = 0.10), NHP total score (r = 0.039, *p* = 0.85), NHP pain (r = 0.232, *p* = 0.25), NHP emotional reactions (r = −0.118, *p* = 0.57), NHP social isolation (r = −0.132, *p* = 0.52), NHP physical activity (r = 0.104, *p* = 0.61), and NHP energy (r = 0.086, *p* = 0.69) scores with the length of ICU stay. A correlation was found between the APACHE II scores with vaccination status (r = −0.426, *p* < 0.03), percent predicted FVC (r = −0.455, *p* = 0.02), 6MWT distance (r = −0.449, *p* = 0.02), the change in SpO_2_ (r = 0.417, *p* = 0.03), HADS (r = 0.394, *p* = 0.046), SGRQ activity (r = 0.396, *p* = 0.045), and SGRQ impact (r = 0.428, *p* = 0.02) scores. However, no significant correlation was found between TUG (r = 0.015, *p* = 0.94), MMRC (r = 0.368, *p* = 0.06), percent predicted FEV_1_ (r = −0.315, *p* = 0.11), handgrip strength (r = −0.330, *p* = 0.10), NHP total score (r = 0.131, *p* = 0.52), NHP pain (r = 0.169, *p* = 0.41), NHP emotional reactions (r = 0.052, *p* = 0.80), NHP sleeping (r = 0.122, *p* = 0.55), NHP social isolation (r = −0.120, *p* = 0.55), NHP physical activity (r = 0.259, *p* = 0.20), NHP energy (r = 0.142, *p* = 0.49), SGRQ symptom (r = 0.372, *p* = 0.06), and SGRQ total score (r = 0.372, *p* = 0.06) with APACHE II scores. A correlation was found between the TUG scores with percent predicted FVC (r = −0.442, *p* = 0.02), percent predicted FEV_1_ (r = −0.546, *p* = 0.004), NHP total score (r = 0.494, *p* = 0.01), NHP emotional reactions (r = 0.418, *p* = 0.03), NHP sleeping (r = 0.555, *p* = 0.003), and NHP physical activity (r = 0.490, *p* = 0.01) scores. However, no significant correlation was found between vaccination status (r = 0.298, *p* = 0.14), MMRC (r = 0.221, *p* = 0.28), 6MWT distance (r = −0.075, *p* = 0.71), the change in SpO_2_ (r = −0.242, *p* = 0.23), handgrip strength (r = −0.295, *p* = 0.14), HADS (r = 0.102, *p* = 0.62), NHP pain (r = 0.146, *p* = 0.48), NHP social isolation (r = −0.030, *p* = 0.89), and NHP energy (r = 0.353, *p* = 0.08) scores with the TUG.

### 3.10. Laboratory Changes in Patients with COVID-19

The laboratory changes in patients of COVID-19 are summarized in Table 5. White blood cell (WBC), neutrophil (NE), lymphocyte (LY), and platelet (PLT) values did not change; however, biochemical, coagulation, and inflammatory biomarkers all increased. In addition, although the mean values of WBC and LY were within the reference value range, it was found that WBC values were above the reference values in 8 of the patients, and LY values were below the reference values in 10 of them.

### 3.11. Relationship between CRP and LDH with FVC, 6MWT Distance, Dyspnea, and SGRQ Total Score

**Figure 4:**
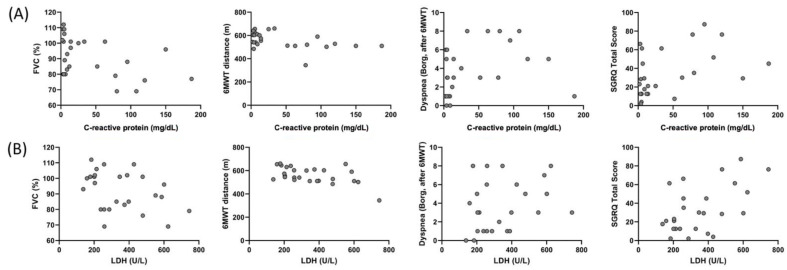
Correlation of the C-reactive protein with (**A**) FVC (%) (r = −0.392, *p* = 0.048), 6MWT distance (r = −0.460, *p* = 0.01), dyspnea (Borg, after 6MWT) (r = 0.417, *p* = 0.03), and SGRQ total score (r = 0.465, *p* = 0.01), and correlation of LDH with (**B**) FVC (%) (r = −0.406, *p* = 0.04), 6MWT distance (r = −0.516, *p* = 0.007), dyspnea (Borg, after 6MWT) (r = 0.321, *p* = 0.11), and SGRQ total score (r = 0.442, *p* = 0.02) in the COVID-19 group.

Correlation of CRP and LDH of the individuals in the COVID-19 group with FVC (%), 6MWT distance, dyspnea (after 6MWT), and SGRQ total score are shown Figure 4. A correlation was found between CRP and FVC (%) (r = −0.392, *p* = 0.048), 6MWT distance (r = −0.460, *p* = 0.01), dyspnea (Borg, after 6MWT) (r = 0.417, *p* = 0.03), and SGRQ total score (r = 0.465, *p* = 0.01), and between LDH and FVC (%) (r = −0.406, *p* = 0.04), 6MWT distance (r = −0.516, *p* = 0.007), and SGRQ total score (r = 0.442, *p* = 0.02) in the COVID-19 group.

### 3.12. Relationship between SGRQ Total Score and NHP Total Score with Dyspnea (Borg, after 6MWT), HADS, and FSS

**Figure 5:**
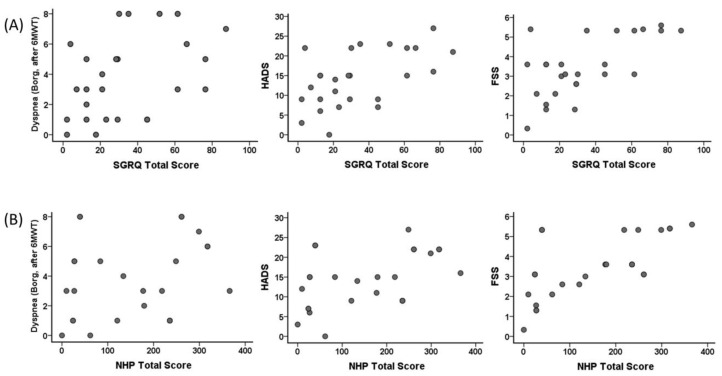
Correlation of the St. George Respiratory Questionnaire (SGRQ) total score with (**A**) dyspnea (Borg, after 6MWT) (r = 0.448, *p* = 0.02), Hospital Anxiety and Depression Scale (HADS) (r = 0.543, *p* = 0.004), and Fatigue Severity Scale (FSS) (r = 0.528, *p* = 0.006), and correlation of Nottingham Health Profile (NHP) total score with (**B**) dyspnea (Borg, after 6MWT) (r = 0.358, *p* = 0.07), HADS (r = 0.536, *p* = 0.005), and FSS (r = 0.723, *p* < 0.001) in the COVID-19 group.

Correlation of SGRQ total score and NHP total score of the individuals in the COVID-19 group with dyspnea (Borg, after 6MWT), HADS, and FSS are shown in Figure 5. A correlation was found between SGRQ total scores and dyspnea (Borg, after 6MWT) (r = 0.448, *p* = 0.02), HADS (r = 0.543, *p* = 0.004), and FSS (r = 0.528, *p* = 0.006). A correlation was found between NHP total score and HADS (r = 0.536, *p* = 0.005) and FSS (r = 0.723, *p* < 0.001). However, no significant correlation was found between dyspnea (Borg, after 6MWT) (r = 0.358, *p* = 0.07) and NHP total score.

## 4. Discussion

In this paper, we have shown respiratory dysfunction in COVID-19 patients after 6 months of discharge from ICU. Findings favored fibrosis in the lung, low oxygen saturation, desaturation during 6MWT, increased shortness of breath, fatigue, and depression and decreased functional capacity, peripheral muscle strength, quality of life, and balance in individuals with severe COVID-19 even 6 months after recovering from the infection. In addition, we found that length of ICU stay and APACHE II scores were associated with FVC and FEV_1_, 6MWT distance, change in SpO_2_, handgrip strength, HADS, and SGRQ total score. In line with these results, we believe that the lungs of individuals with severe COVID-19 have permanent damage that reduces respiratory function, functional capacity, and quality of life.

The inflammatory biomarkers return to typical values in the third month of post-COVID, but lung function gradually deteriorates [11] with consolidation, fibrosis, and interstitial pattern in CT scans taken 3 months later [11]. We found lower FVC, FEV_1_, PEF, and FEF_25–75%_ of the individuals with COVID-19. FVC and FEV_1_ values of nine and seven individuals, respectively, were below the LLN stating impaired respiratory function in the COVID-19 group. The higher FEV_1_/FVC values despite decreased FEV_1_ and FVC (although not statistically significant, *p* = 0.25) suggested the development of a restrictive type of lung disease in patients with COVID-19. Together with similar respiratory muscle strength between COVID-19 patients and controls, decreased lung volume was not associated with respiratory muscle weakness expected due to the stay in the ICU. Considering the decrease in all measured volume values, a restrictive type of lung disease may have developed [29] since pneumonia is almost completely cleared from the lung tissue within 5–6 months, and fibrous tissue is formed in this process [12].

Ferrandi et al. [30] argue that some skeletal muscle types may show vulnerability to the COVID-19 virus through angiotensin-converting enzyme-2 (ACE-2). The coronavirus is active in the lungs, the leukocytes infiltrating the lung tissue, and the cytokines (especially IL-6) are secreted by these leukocytes. This disrupts metabolic hemostasis and causes muscle loss by infiltrating into the muscle [30]. In our study, the handgrip and quadriceps muscle strength were lower in the COVID-19 group, which can be attributed to the above mechanism explaining muscle loss. In addition, insufficient oxygen diffusion and transport to peripheral tissues due to fibrosis might explain peripheral muscle weakness.

Pathophysiological changes resulting from ARDS are associated with COVID-19 [31]. Secondary lung injury such as edema, pulmonary inflammation, abnormal surfactant function, decrease in compliance, and deterioration in gas diffusion occur due to prolonged IMV, and these changes may decrease the functional capacity [31]. We found impaired functional capacity measured using 6MWT and peripheral muscle strength in severe COVID-19 patients 6 months after discharge. The loss in peripheral muscle strength may have resulted in the decreased 6MWT distance. Six individuals in the COVID-19 group were desaturated during the test. The changes in SpO_2_ may suggest that reduced 6MWT distance is primarily due to possible lung fibrosis in the COVID group, as demonstrated in post-COVID-19 CTs [11].

Inflammation and inflammatory cytokines may disrupt the balance by causing damage to the cerebellar tissue [32]. Rudroff et al. showed impaired balance in the COVID-19 patients based on the TUG and the stabiliometer findings [32]. A balance disorder may be due to immobilization restriction during the stay in the ICU. However, we found no correlation between the length of stay in the ICU and TUG results. Therefore, it is difficult to say that the loss of balance is due to immobilization. TUG was associated with NHP and FVC and FEV_1_. In other words, inflammation and inflammatory cytokines may be related to the damage in cerebellar tissue, as stated by Rudroff et al. [33], and TUG being associated with respiratory parameters indirectly indicates the relationship of balance with inflammation. Further study is needed to identify the mechanisms of balance disorder, including the neuromuscular and psychological covariates in COVID-19 patients.

The factors likely to cause fatigue in COVID-19 are central, psychological, and peripheral [33]. Psychological problems in individuals who have survived the COVID-19 infection are considered to be part of post-traumatic stress disorder [32,34]. Fears such as being exposed to the virus again, being in ICU, and losing relatives cause psychological trauma. In this direction, we evaluated the HADS scores and found higher scores in the COVID-19 group. Hospitalization due to COVID and the strong correlation between the length of stay in the ICU and HADS scores support the idea of post-traumatic stress disorder [34]. Central factors may include penetration of coronaviruses into the central nervous system triggering inflammation by affecting the release of neurotransmitters, and causing permanent problems such as fatigue due to cerebral hypometabolism. Prolonged physical inactivity due to the disease and ICU stay may be one of the factors causing fatigue [33]. We found higher MMRC, FSS, respiratory frequency, general fatigue, leg fatigue, and dyspnea scores after 6MWT in the COVID-19 group. There were high fatigue levels (general fatigue and leg fatigue before 6MWT and FSS findings) even before the test (at rest). The increase in fatigue after 6MWT and the presence of desaturated individuals during the test showed possible gas diffusion problems and central factors [33]. The individuals in the COVID-19 group may not carry enough oxygen to their active muscles during exercise, and the severity of fatigue increases.

Based on the SGRQ and NHP scores, the quality of life of individuals in the COVID-19 group was impaired. From the activity and physical component subscale scores in the COVID-19 patients, one of the critical factors that reduced the quality of life of these individuals may be tiredness. Brugge et al. [35] evaluated COVID-19 patients 6 weeks after discharge and found a decrease in quality of life which is related to diffusion capacity [35]. Considering the presence of desaturation with lower SpO_2_ values in the COVID-19 group, the quality of life may be affected by the decrease in diffusion capacity, similar to the study of Brugge et al. Similarly, high MMRC and Borg Scale dyspnea scores before and after 6MWT in the COVID-19 group may explain impaired quality of life in this group.

Corticosteroid treatment is administered to individuals with severe COVID-19 infection, especially those with cytokine storm, that is, severe inflammation [36]. The corticosteroid treatment reduces the length of stay in ICU and the mortality rate [37]. In the present study, FVC, FEV_1_, 6MWT distance, and SpO_2_ (after 6MWT) values of the patients who received corticosteroid treatment and were followed up with IMV were lower, and the FEV_1_/FVC value was higher as compared to those who did not (Table 3). In addition, the longer length of stay in the ICU and higher APACHE II scores in those receiving corticosteroid therapy also prove that respiratory function loss is associated with the severe course of the disease (Table 3). In other words, individuals with a more severe infection received corticosteroid treatment and were followed up with IMV. Accordingly, due to the pathological changes, lung compliance, diffusion capacity, and respiratory function decreases more in those with severe COVID-19 infection.

Specific blood laboratory findings are seen in patients with severe COVID-19 [38]. A meta-analysis stated that in patients with severe COVID-19, hemoglobin decreased especially due to hemoglobin destruction resulting increased ferritin and D-dimer due to iron emerging. Ferritin is a marker that shows inflammatory load together with C-reactive protein (CRP) and erythrocyte sedimentation rate (ESR) [38]. Similarly, we observed lower HBG and higher D-dimer values (Table 5). CRP and lactate dehydrogenase (LDH) values are associated with inflammation and inflammation-associated cellular damage [38]. More importantly, the finding that CRP, a strong inflammatory marker, was associated with FVC, 6MWT distance, dyspnea, and SGRQ total score (Figure 4) indicated that inflammation in the lung affects respiratory function and functional capacity, even after 6 months of discharge. Similarly, LDH, which is an indicator of cellular damage, was also found to be associated with FVC, 6MWT distance, and SGRQ total score.

We found a relationship between FVC, FEV_1_, the change in SpO_2_, handgrip strength, HADS, SGRQ values, and 6MWT distance with the length of stay in ICU even 6 months after discharge. One of the critical points is the SpO_2_ value. The association of the change in SpO_2_ with ICU stay is probably due to the severe disease course and pathological processes. The correlation between SGRQ activity and SGRQ impact with APACHE II scores showed that the quality of life parameters of individuals with severe infection was significantly affected by the dimensions associated with physical activity after 6 months post-COVID-19 infection. The association of SGRQ and NHP total scores with dyspnea (Borg score after 6MWT), HADS, and FSS indicated that the continued decline in quality of life even after 6 months post-COVID is associated with increased fatigue, dyspnea, and depression (Figure 4).

Some limitations should be noted when interpreting the findings of this study. Pre-COVID-19 respiratory function, functional capacity, fatigue levels, and quality of life of the individuals were unknown. In that case, the change in these parameters during the process could be determined more clearly. We could have strengthened our obtained data if we had evaluated the disease severity score and had the diffusion capacity and lung CT data 6 months after discharge. Apart from these, it is the first study to functionally evaluate patients 6-months post-COVID.

In the literature, it is generally stated that fibrosis may develop in the lung tissue of patients after COVID-19, based on CT and blood laboratory results. In this study, we tried to prove the hypotheses put forward by studies in the literature with more functional evaluations. In future studies, fibrosis in the lung tissue can be evaluated using more objective evaluation methods, and its relations with similar functional parameters in this study can be investigated. In addition, whatever the underlying factor is, it is an undeniable fact that low oxygen saturation, increased shortness of breath, fatigue, decreased functional capacity, peripheral muscle strength, quality of life and balance, and depression are seen in these patients. Therefore, it should be considered that these patients may be good candidates for pulmonary rehabilitation in previous studies. Studying the effectiveness of pulmonary rehabilitation will make valuable contributions to the literature.

## 5. Conclusions

From our results, we conclude that patients with COVID-19 infection followed in the ICU due to pulmonary inflammation (indicated by markers of inflammation and cell damage such as CRP and LDH), consolidation, edema, and ARDS who need oxygen support and IMV continue to suffer from loss of respiratory function and decreased muscle strength, functional capacity, dyspnea, oxygen saturation, balance, quality of life, and fatigue even 6 months after discharge. The prolonged symptoms are probably due to fibrosis in the lung tissue during the healing process and the developing interstitial disease. In terms of early intervention for possible interstitial lung disease, it may be essential to follow the pulmonary function tests for at least 6 months in the post-discharge period in patients infected with COVID-19. In addition, these patients’ functional capacity and physiological and perceptional characteristics, primarily their fatigue levels, should be evaluated, and COVID-19 patients should be considered for inclusion in a pulmonary rehabilitation program in the early period.

## Figures and Tables

**Table 1:** Physical and demographic characteristics and dyspnea in COVID-19 and control groups.

Parameter ^a^	COVID-19 Group (*n* = 26)	Control Group (*n* = 26)	*p*
	Mean ± SD	Mean ± SD	
Age (years)	50.0 ± 13.8	47.0 ± 12.6	0.41
Height (cm)	171.2 ± 9.3	171.0 ± 7.0	0.89
Weight (kg)	79.1 ± 15.5	73.8 ± 13.8	0.20
BMI (kg/m^2^)	27.1 ± 5.9	25.1 ± 3.7	0.15
MMRC score	0.6 ± 0.5	0.1 ± 0.3	<0.001 *
Smoking (pack-years)	19.2 ± 15.3	20.0 ± 8.1	0.90
Number of active smokers	10	7	

* The bold values represent statistically significant *p* < 0.05 values. ^a^ Student’s *t*-test. BMI—Body Mass Index; MMRC—Modified Medical Research Council Dyspnea Scale.

**Table 2:** Comparison of MMST, HADS, FSS, TUG, physical activity, and quality of life in COVID-19 and control groups.

Variables	COVID-19 Group (*n* = 26)	Control Group (*n* = 26)	*p*
	Median (Min-Max)	Median (Min-Max)	
HADS ^a,c^	14.2 ± 7.0	5.9 ± 3.6	<0.001 *
FSS ^a,c^	3.5 ± 1.5	1.2 ± 0.7	<0.001 *
MMST total score ^a,c^	27.7 ± 2.8	28.2 ± 1.5	0.42
TUG ^a,c^	7.9 ± 1.2	7.2 ± 0.9	0.049 *
International Physical Activity Questionnaire ^b^			
High intensity (MET-min/week)	0 (0–3360)	0 (0–30240)	0.10
Medium intensity (MET-min/week)	0 (0–3600)	240 (0–1440)	0.23
Walking score (MET-min/week)	1386 (132–2772)	792 (0–11088)	0.14
Sitting score (MET-min/week)	5 (3–6)	5 (4–6)	0.99
Total score (MET-min/week)	1391 (136–6736)	929 (324–41333)	0.08
St George Respiratory Questionnaire ^b^			
Symptom	30.5 (0–69.7)	14.0 (0–40.4)	0.01 *
Activity	38.0 (0–95.6)	0 (0–62.9)	<0.001 *
Impact	16.7 (0–78.1)	4.0 (0–15.0)	0.001 *
Total Score	28.9 (2.1–87.4)	9.7 (0–30.0)	0.001 *
Nottingham Health Profile ^b^			
Pain	18.6 (0–89.5)	0 (0–27.4)	0.01 *
Emotional Reactions	10.5 (0–92.8)	0 (0–31.5)	0.02 *
Sleep	19.9 (0–61.5)	0 (0–77.6)	0.42
Social Isolation	0 (0–77.5)	0 (0–16.0)	0.03 *
Physical Activity	16.9 (0–41.9)	0 (0–22.0)	<0.001 *
Energy	24 (0–100.0)	0 (0–36.8)	<0.001 *
Total Score	155.6 (0–366.0)	30.6 (0–130.9)	0.001 *

* The bold values represent statistically significant *p* < 0.05 values. ^a^ Student t-test. ^b^ Mann–Whitney U test (different tests were used due to distribution difference). ^c^ Expressed in mean ± SD. HADS—Hospital Anxiety and Depression Inventory; FSS—Fatigue Severity Scale; MMST—Mini-mental State Test; TUG—Time-Up Go Test.

**Table 3:** Comparison of respiratory function and functional capacity of individuals with COVID-19 between those who received and did not receive steroid therapy and those who received NIMV and IMV.

Parameter	Receiving Steroid Therapy (*n* = 15)	Not Receiving Steroid Therapy (*n* = 11)	*p*	NIMV (*n* = 12)	IMV (*n* = 14)	*p*
	Median (Min-Max)	Median (Min-Max)		Median (Min-Max)	Median (Min-Max)	
FVC (%) ^b^	83.0 (69.0–102.0)	101.0 (89.0–112.0)	0.001 *	101.0 (80.0–112.0)	84.0 (69.0–102.0)	0.004 *
FEV_1_ (%) ^b^	90.0 (78.0–108.0)	103.0 (87.0–113.0)	0.008 *	100.0 (81.0–113.0)	90.0 (78.0–108.0)	0.04 *
FEV_1_/FVC ^b^	105.0 (101.0–123.0)	101.0 (94.0–113.0)	0.01 *	101.0 (94.0–113.0)	106.5 (101.0–123.0)	0.02 *
6MWT distance (m) ^a,c^	532.7 ± 68.5	599.9 ± 56.2	0.01 *	603.2 ± 54.8	525.1 ± 64.1	0.003 *
SpO_2_ (after 6MWT) (%) ^a,c^	92.0 (82.0–96.0)	95.0 (90.0–97.0)	0.04 *	95.5 (90.0–97.0)	91.0 (82.0–96.0)	0.01 *
Length of stay in the ICU (days) ^a,c^	9.9 ± 7.9	4.1 ± 3.9	0.02 *	3.8 ± 3.8	10.5 ± 7.8	0.01 *
APACHE II ^a,c^	15.5 ± 7.0	10.1 ± 5.5	0.046 *	9.9 ± 5.3	16 ± 6.9	0.02 *

* The bold values represent statistically significant *p* < 0.05 values. ^a^ Student t-test. ^b^ Mann–Whitney U test (different tests were used due to distribution difference). ^c^ Expressed as mean ± SD. FVC—Forced vital capacity; FEV_1_—Forced expiratory volume in one second; 6MWT—6-minute walk test; SpO_2_—Oxygen saturation.

**Table 4:** Correlation of the length of stay in the intensive care unit and APACHE II scores of the individuals in the COVID-19 group with the measured parameters.

Parameter ^d^	Length of Stay in the Intensive Care Unit		APACHE II Scores		Time Up Go Test	
	r	*p*	r	*p*	r	*p*
Vaccination status	−0.633	0.001 *	−0.426	0.03 *	0.298	0.14
Time Up Go Test	−0.095	0.64	0.015	0.94	-	-
MMRC	0.327	0.10	0.368	0.06	0.221	0.28
FVC	−0.564	0.003 *	−0.455	0.02 *	−0.442	0.02 *
FEV_1_	−0.453	0.02 *	−0.315	0.12	−0.546	0.004 *
6MWT distance	−0.427	0.03 *	−0.449	0.02 *	−0.075	0.72
ΔSpO_2_	0.469	0.02 *	0.417	0.03 *	−0.242	0.23
Handgrip strength	−0.487	0.01 *	−0.330	0.10	−0.295	0.14
HADS	0.394	0.047 *	0.394	0.046 *	0.102	0.62
NHP Total Score	0.039	0.85	0.131	0.52	0.494	0.01 *
NHP Pain	0.232	0.25	0.169	0.41	0.146	0.48
NHP Emotional Reactions	−0.118	0.57	0.052	0.80	0.418	0.03 *
NHP Sleep	0.018	0.93	0.122	0.55	0.555	0.003 *
NHP Social Isolation	−0.132	0.52	−0.120	0.56	−0.030	0.89
NHP Physical Activity	0.104	0.61	0.259	0.20	0.490	0.01 *
NHP Energy	0.086	0.67	0.142	0.49	0.353	0.08
SGRQ Total Score	0.568	0.002 *	0.372	0.06	0.112	0.59
SGRQ Symptom	0.551	0.004 *	0.339	0.09	0.101	0.62
SGRQ Activity	0.532	0.005 *	0.396	0.045 *	0.133	0.52
SGRQ Impact	0.573	0.002 *	0.428	0.03 *	0.074	0.72

* The bold values represent statistically significant *p* < 0.05 values. ^d^ Spearman correlation analysis. Vaccination status: Number of vaccine doses of the individuals. MMRC—Modified Medical Research Council; FVC—Forced vital capacity; FEV_1_—Forced expiratory volume in one second; 6MWT—6-minute walk test; ΔSpO_2_—Oxygen saturation difference before and after 6MWT; HADS—Hospital Anxiety and Depression Scale; NHP—Nottingham Health Profile; SGRQ—St. George Respiratory Questionnaire.

**Table 5:** Summary of laboratory findings in patients with COVID-19.

Parameter	Mean ± SD	Changes with Reference Value
Hematologic		
White blood cell count (10^3^/µL)	8.4 ± 3.6	↔
Neutrophil (%)	61.8 ± 10.5	↔
Lymphocyte (%)	18.1 ± 11.5	↔
Hemoglobin (g/dL)	11.0 ± 2.3	↓
Hematocrit (%)	37 ± 4.5	↓
Platelet count (10^3^/µL)	201.7 ± 65.6	↔
Biochemical		
Lactate dehydrogenase (U/L)	350.4 ± 167.2	↑
Creatine kinase-MB (U/L)	33.2 ± 12.9	↑
Aspartate aminotransferase (U/L)	62.9 ± 51.8	↑
Alanine aminotransferase (U/L)	59.0 ± 56.7	↑
Troponin T (ng/L)	30.1 ± 33.4	↑
Kreatinin (mg/dL)	1.4 ± 0.8	↑
Coagulation		
Prothrombin time (sn)	16.8 ± 2.4	↑
D-dimer (µg/L)	829.9 ± 1027.5	↑
Inflammatory biomarkers		
Ferritin (µg/L)	343.5 ± 184.4	↑
C-reactive protein (mg/L)	42.3 ± 46.5	↑
Erythrocyte sedimentation rate (mm/h)	16.8 ± 8.8	↑

Changes with reference value: ↔ no change, ↑ increase, ↓ decrease.

## Data Availability

Data from this study are available from the corresponding author on reasonable request.
